# An epitope tag alters phosphoglycerate dehydrogenase structure and impairs ability to support cell proliferation

**DOI:** 10.1186/s40170-015-0131-7

**Published:** 2015-04-29

**Authors:** Katherine R Mattaini, Edward J Brignole, Mitali Kini, Shawn M Davidson, Brian P Fiske, Catherine L Drennan, Matthew G Vander Heiden

**Affiliations:** Koch Institute for Integrative Cancer Research, Massachusetts Institute of Technology, Cambridge, MA 02139 USA; Department of Biology, Massachusetts Institute of Technology, Cambridge, MA 02139 USA; Department of Chemistry Massachusetts Institute of Technology, Cambridge, MA 02139 USA; Howard Hughes Medical Institute Massachusetts Institute of Technology, Cambridge, MA 02139 USA; Dana-Farber Cancer Institute, Boston, MA 02215 USA; Broad Institute, Cambridge, MA 02139 USA

**Keywords:** Phosphoglycerate dehydrogenase, Serine metabolism, Serine synthesis, Cancer Metabolism, Epitope tag

## Abstract

**Background:**

The gene encoding the serine biosynthesis pathway enzyme PHGDH is located in a region of focal genomic copy number gain in human cancers. Cells with *PHGDH* amplification are dependent on enzyme expression for proliferation. However, dependence on increased PHGDH expression extends beyond production of serine alone, and further studies of PHGDH function are necessary to elucidate its role in cancer cells. These studies will require a physiologically relevant form of the enzyme for experiments using engineered cell lines and recombinant protein.

**Results:**

The addition of an N-terminal epitope tag to PHGDH abolished the ability to support proliferation of *PHGDH*-amplified cells despite retention of some activity to convert 3-PG to PHP. Introducing an R236E mutation into PHGDH eliminates enzyme activity, and this catalytically inactive enzyme cannot support proliferation of *PHGDH-*dependent cells, arguing that canonical enzyme activity is required. Tagged and untagged PHGDH exhibit the same intracellular localization and ability to produce D-2-hydroxyglutarate (D-2HG), an error product of PHGDH, arguing that neither mislocalization nor loss of D-2HG production explains the inability of epitope-tagged PHGDH to support proliferation. To enable studies of PHGDH function, we report a method to purify recombinant PHGDH and found that untagged enzyme activity was greater than N-terminally tagged enzyme. Analysis of tagged and untagged PHGDH using size exclusion chromatography and electron microscopy found that an N-terminal epitope tag alters enzyme structure.

**Conclusions:**

Purification of untagged recombinant PHGDH eliminates the need to use an epitope tag for enzyme studies. Furthermore, while tagged PHGDH retains some ability to convert 3PG to PHP, the structural alterations caused by including an epitope tag disrupts the ability of PHGDH to sustain cancer cell proliferation.

**Electronic supplementary material:**

The online version of this article (doi:10.1186/s40170-015-0131-7) contains supplementary material, which is available to authorized users.

## Background

**Figure 1:**
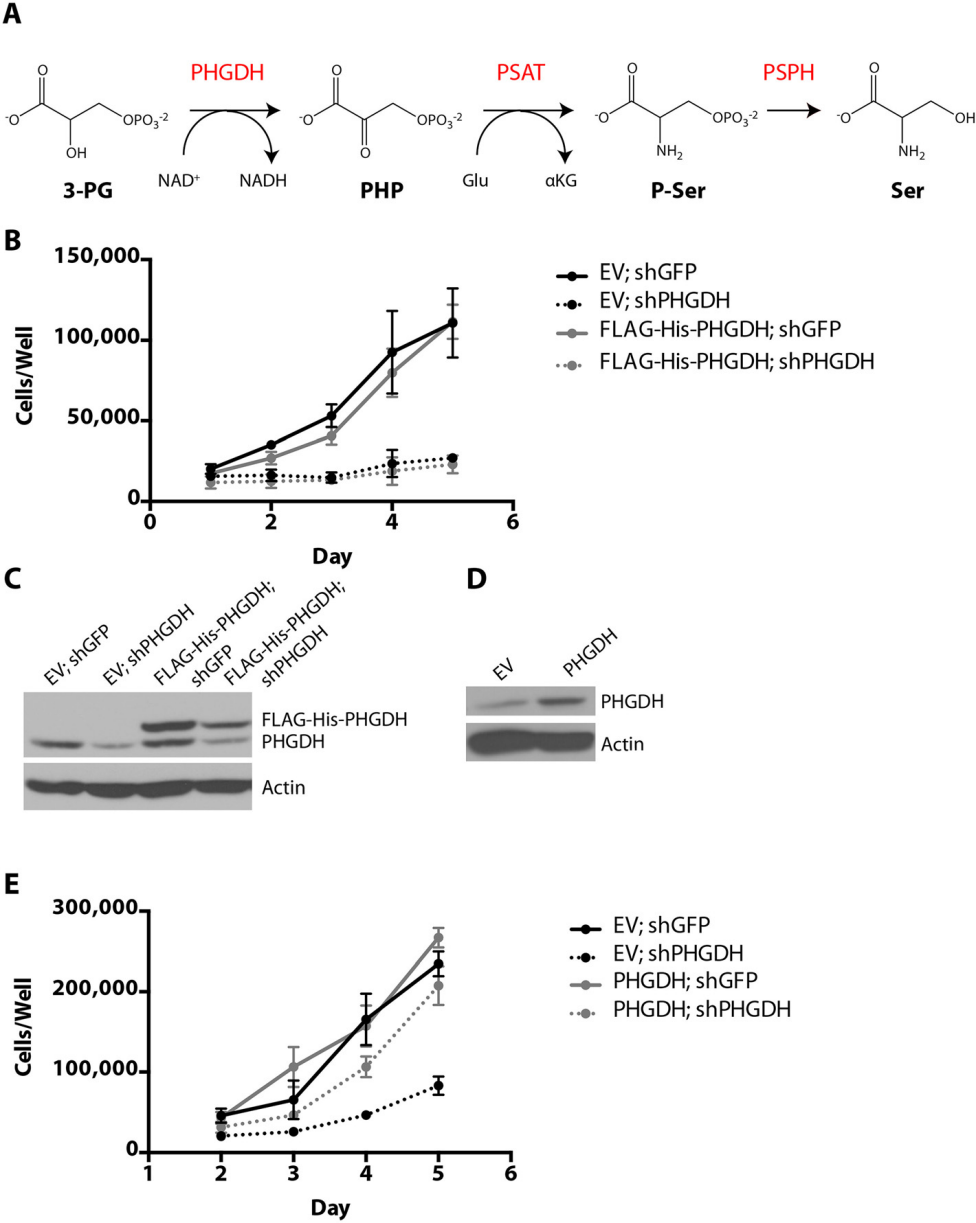
**N-terminal epitope-tagged PHGDH cannot support cell proliferation following PHGDH knockdown. (A)** Schematic representation of the serine biosynthesis pathway. 3-PG, 3-phosphoglycerate; PHGDH, 3-phosphoglycerate dehydrogenase; NAD^+^/NADH, oxidized and reduced forms of nicotinamide adenine dinucleotide, respectively; PHP, phosphohydroxypyruvate; PSAT, phosphoserine aminotransferase; Glu, glutamate; αKG, alpha-ketoglutarate; P-Ser, phosphoserine; PSPH, phosphoserine phosphatase; Ser, serine. **(B)** Cell number over time of *PHGDH*-amplified T.T. cells stably expressing either an shRNA-resistant FLAG-His-PHGDH cDNA or empty vector (EV) control. **(C)** Western blot analysis assessing knockdown of endogenous PHGDH and expression of FLAG-His-PHGDH cDNA. **(D)** Western blot analysis of T.T. cells stably expressing an shRNA-resistant PHGDH cDNA (untagged) or empty vector (EV) control. **(E)** Cell number over time of the cells described in (D) when infected with virus expressing GFP or PHGDH shRNA. Error bars show standard deviation from the mean.

Flux through the pathway that synthesizes serine from glucose is important for proliferation of some cancer cells. D-3-phosphoglycerate dehydrogenase (PHGDH) catalyzes the first reaction in serine biosynthesis. Using NAD^+^ as a cofactor, PHGDH oxidizes the glycolytic intermediate 3-phosphoglycerate (3-PG) to phosphohydroxypyruvate (PHP) (Figure 1A). Subsequent steps in serine biosynthesis involve phosphoserine aminotransferase 1 (PSAT1)-catalyzed transamination of PHP and glutamate to phosphoserine (P-Ser) and alpha-ketoglutarate (αKG), followed by phosphoserine phosphatase (PSPH)-mediated dephosphorylation of P-Ser to produce serine. Serine has multiple fates in cells, serving as an amino acid for protein synthesis and a precursor for lipid head groups. Serine can also be further metabolized to glycine by serine hydroxymethyltransferase, which donates a one-carbon unit to the folate pool for nucleotide synthesis and methyltransferase reactions. In addition to its role as an amino acid, glycine also contributes to nucleotide synthesis and glutathione production [1]. In this way, the biosynthesis of serine from glucose plays a central role in cancer cell metabolism.

PHGDH expression is important in human cancers. *PHGDH* is located in a region of genomic copy number gain that includes four other genes in the smallest common amplified region [2]. *PHGDH* copy number gain has been observed with the highest frequency in melanoma and breast cancer, and *PHGDH*-amplified cell lines are dependent on enzyme expression to proliferate in culture [3,4]. Increased expression of PHGDH and other serine biosynthesis pathway enzymes is found in other cancer contexts as well, with evidence that c-Myc [5], ATF4 [6], and the lysine methyltransferase G9A [7] increase PHGDH expression via transcriptional and epigenetic mechanisms. Increased activity of serine biosynthesis pathway enzymes has been hypothesized to support increased nucleotide biosynthesis in cancer [8], although cell lines vary in their dependence on PHGDH expression for growth as xenograft tumors [4,9].

Studies have also demonstrated that exogenous serine is critical for tumor cell proliferation *in vitro* and as xenografts [10-12]; however, there may be a distinct requirement for *de novo* serine synthesis from glucose even in the presence of abundant serine. Exogenous serine does not rescue proliferation of *PHGDH*-amplified cells following knockdown of PHGDH expression [4,9]; moreover, the expression of PSAT and PSPH is also required for the continued proliferation of at least some of these cells [3]. Taken together, these data suggest that flux through the serine biosynthesis pathway is important for cell metabolism beyond providing serine. One possibility is a role in TCA cycle anapleurosis [4], but it remains controversial whether this underlies the requirement for PHGDH in all cells [2,3,13].

Interrogating PHGDH function(s) would benefit from studies of purified enzyme. Past comprehensive studies of PHGDH enzyme activity examined the *Escherichia coli* protein [14-17], which has a different domain structure than the human enzyme [18]. Structural studies of the human enzyme are limited to a truncated protein (PDB 2G76, unpublished), and enzymatic analysis often uses epitope-tagged protein [19,20]. While epitope tags are commonly used to isolate recombinant enzyme, in some cases, these tags can alter structure and/or function [21]. Therefore, both an understanding of whether an epitope tag will impact PHGDH function and methods to produce recombinant enzyme that reflects endogenous protein activity in cells are necessary to determine the role of PHGDH in human cancer and normal tissues.

We observed that, unlike untagged protein, the expression of PHGDH with an epitope tag was unable to rescue cell proliferation following PHGDH knockdown in *PHGDH*-amplified cells. An enzymatically dead PHGDH mutant also failed to rescue proliferation of cells following PHGDH knockdown, suggesting that enzyme activity is required for cells to proliferate. We present a method for purification of untagged recombinant PHGDH and find that the activity of this protein is appreciably greater than recombinant PHGDH with an N-terminal epitope tag. Finally, we show using size exclusion chromatography and electron microscopy that an N-terminal epitope tag on PHGDH affects protein structure, which may contribute to an inability to substitute for endogenous PHGDH in cells. These data argue that the use of epitope tags should be avoided in future studies of PHGDH function.

## Methods

### Cell culture, viral infection, and assessment of cell proliferation

Cells were cultured in DMEM supplemented with 10% FBS, penicillin, and streptomycin. We stably introduced shRNA-resistant PHGDH cDNAs into cells using the retroviral vector pLHCX and selected with 150 μg/ml hygromycin. To decrease PHGDH expression, shRNA construct TRCN0000028548 with target sequence 5′-AGGTGATAACACAGGGAACAT-3′ was used. Cells were infected with lentivirus to express shRNAs, selected for 3 days in 1 μg/ml puromycin and proliferation assessed on subsequent days.

### Western blot analysis

PHGDH protein was detected using a rabbit polyclonal anti-PHGDH antibody from Sigma, St. Louis, MO, USA (HPA021241) at 0.2 μg/ml and actin detected using a rabbit polyclonal anti-actin antibody from Abcam, Cambridge, MA, USA (ab1801) at 1 μg/ml, appropriate HRP-conjugated secondary antibodies and chemiluminescence.

### Site-directed mutagenesis

The PHGDH R236E mutant was generated using the QuickChange II XL Site-Directed Mutagenesis Kit from Agilent, Santa Clara, CA, USA, with the following primers: 5′-TGTGGTGAACTGTGCCGAGGGAGGGATCGTGGACG-3′ and 5′-CGTCCACGATCCCTCCCTCGGCACAGTTCACCACA-3′. To generate shRNA-resistant PHGDH, we used the same kit and the following primers: 5′-CCCAAAGGGACCATCCAAGTTATCACACAGGGAACATCCC-3′ and 5′-GGGATGTTCCCTGTGTGATAACTTGGATGGTCCCTTTGGG-3′.

### Purification of epitope-tagged PHGDH and PSAT proteins

pTriEx-3 FLAG-His-PHGDH (wild type or R236E mutant) or pET28a His-PHGDH was transformed into BL21 (DE3) *E. coli*. Single colonies from these plates were inoculated into LB plus ampicillin (pTriEx-3) or kanamycin (pET28a) and grown at 37°C and 225 rpm overnight. The next day, cultures were diluted 1:40 and grown to OD_600_ = 0.4, and then 0.5 mM IPTG was added before incubating at 16°C and 225 rpm overnight. The bacteria were pelleted at 6,000 × *g* for 15 min, and the pellet was flash frozen and stored at −80°C. All protein purification steps were performed at 4°C. To purify protein, bacterial pellets were resuspended in 30 ml lysis buffer (50 mM Tris, pH 8.5, 10 mM MgCl_2_, 300 mM NaCl, 10% glycerol, and 5 mM imidazole) and sonicated, and the lysate was centrifuged at 20,000 × *g* for 45 min. β-Mercaptoethanol (30 μl) was added and the lysate mixed with 3 ml of Ni-NTA agarose (Qiagen, Hilden, Germany) in wash buffer (50 mM Tris, pH 8.5, 10 mM MgCl_2_, 300 mM NaCl, 10% glycerol, and 30 mM imidazole). This mixture was shaken gently for 2 to 3 h, and Ni-NTA beads were pelleted and washed four times with wash buffer. The beads and lysate were transferred to an empty column and protein recovered in elution buffer (50 mM Tris, pH 8.5, 10 mM MgCl_2_, 250 mM NaCl, 10% glycerol, and 250 mM imidazole) with 1-ml fractions collected. Those fractions with high protein content were determined by Bradford assay. Fractions containing appreciable protein were pooled and separated on a GE HiPrep Sephacryl S-200 HR column (GE Healthcare Bio-Sciences, Pittsburgh, PA, USA) using an AKTA FPLC system (GE Healthcare Bio-Sciences, Pittsburgh, PA, USA). The column was run at 0.5 ml/min for one-column volume in storage buffer (20 mM Tris, pH 7.5, 100 mM NaCl, 1 mM TCEP). Fractions were collected from the void volume to the elution volume of a 17-kDa standard. UV absorbance was used to determine those fractions with the highest protein concentration, and those were pooled, 0.5 mM betaine added, and concentrated to 5 to 10 μg/μl purified protein. Fractions from the size exclusion column were later analyzed by SDS-PAGE and Coomassie staining. After destaining, Coomassie-stained gels were imaged on a LiCOR and quantitated by densitometry analysis using ImageJ. Recombinant His-tagged PSAT was generated by an analogous procedure, with differences as follows. pET28a His-PSAT was transformed into BL21 (DE3) *E. coli*, grown as described above to an OD_600_ of 0.7, then 0.5 mM IPTG was added, and the culture was grown at room temperature and 225 rpm for 6 h. After rPSAT was eluted from the Ni-NTA agarose beads, the combined fractions were dialyzed into 50 mM Tris, pH 7.5, 10 mM MgCl_2_, 25 mM NaCl, 20% glycerol, 0.15% beta-mercaptoethanol.

### Preparation of Cibacron Blue F3GA Sepharose

This protocol was modified from [22]. Sixty milliliters of GE Sepharose CL-4B resin (GE Healthcare Bio-Sciences, Pittsburgh, PA, USA) in water was mixed with 8.6 ml of 45 mg/ml Cibacron Blue F3GA (Polysciences, Warrington, PA, USA) for 30 min at room temperature. Twenty-seven milliliters of 2 M sodium sulfate were added, followed by 30 min more mixing at room temperature. Two hundred ninety microliters of 10 M NaOH was added slowly, dropwise with mixing. The resulting bead suspension was mixed at 60°C for 2 h before neutralizing with 15 ml of 2.5 M sodium phosphate buffer, pH 7.5. The beads were washed and then stored in 20% ethanol at 4°C for at least 2 days. Two batches of beads were subsequently mixed and used to fill an empty GE XK 26/40 column (GE Healthcare Bio-Sciences, Pittsburgh, PA, USA) to a column volume of 65 ml and attached to an AKTA FPLC system.

### Purification of untagged PHGDH protein

pET28a PHGDH was transformed into BL21 *E. coli*. From a single colony, a culture was grown to an OD_600_ = 0.4, 0.5 mM IPTG added, and the culture incubated at room temperature and 225 rpm for 6 h. The bacteria were flash frozen, thawed, and resuspended in 50 ml buffer A (50 mM HEPES pH 8.0, 1 mM EDTA) plus 1 mM DTT and protease inhibitors (Roche). This suspension was sonicated and centrifuged at 20,000 × *g* for 45 min, and the supernatant was loaded onto an F3GA column prepared as described above. The column was run at a flow rate of 2 ml/min as follows: 200 ml buffer A, 10-ml linear gradient to 60% buffer B (50 mM HEPES, pH 8.0, 1 mM EDTA, 400 mM KCl), 130 ml 60% buffer B. During the gradient and 60% B wash, 10-ml fractions were collected. Fractions were assessed for the presence of the recombinant protein by UV absorbance, pooled, concentrated, and desalted into buffer A. The resulting solution was fractionated on a GE MonoQ HR 10/10 column (GE Healthcare Bio-Sciences, Pittsburgh, PA, USA) using an AKTA FPLC at a flow rate of 0.5 ml/min as follows: 30 ml buffer A, 45-ml linear gradient to 45% buffer B, 125 ml 45% buffer B. During the 45% B wash, 2-ml fractions were collected. Fractions were assessed for recombinant protein using UV absorbance, pooled and further separated using GE HiPrep Sephacryl S-200 HR column. The column fractions were treated and analyzed as described for epitope-tagged protein to produce the final protein solution.

### Assessment of PHGDH activity

A linked assay was utilized where PHGDH, PSAT, and all substrates were mixed in a well of a 96-well plate and activity assessed at 37°C. The reaction buffer used was 300 mM Tris, pH 9, 1 mM EDTA, 1 mM TCEP, 0.025% BSA. A linked assay with PSAT was used to interrogate 3-PG and NAD+ to PHP and NADH conversion by PHGDH because the equilibrium of this reaction lies toward 3-PG [23]. Enzyme activity was measured by following NADH generation over time by fluorescence, with excitation at 340 nm and emission at 460 nm. All substrates were present in excess of their reported *K*_M_ values (final concentration 10 mM each 3-PG, NAD^+^, and Glu). PSAT was present in excess of PHGDH and control experiments performed to ensure that PSAT was not limiting during the kinetic window evaluated.

### Immunofluorescence and confocal microscopy

Cells were grown on glass coverslips and fixed with 4% paraformaldehyde. Cells were then permeabilized with PBS containing 0.1% Tween 20, blocked in 1% BSA, and incubated in primary antibody overnight at 4°C. Primary antibody was rabbit polyclonal anti-PHGDH antibody from Sigma (HPA021241), used at 0.01 μg/ml. Secondary antibody was AlexaFluor594 donkey anti-rabbit (A21207, Life Technologies, Grand Island, NY, USA) at 2 μg/ml. Cells were counterstained with DAPI (D9542, Sigma, St. Louis, MO, USA) and mounted using Prolong Gold Antifade mounting medium (P36934, Life Technologies, Grand Island, NY, USA). Images were captured using a Deltavision Spectris imaging system (GE Healthcare BioSciences Corp., Piscataway, NJ, USA) with × 100 oil immersion objective and analyzed using *softWoRx* (Applied Precision, Issaquah, WA, USA) and ImageJ (National Institutes of Health) software. Total fluorescence intensity was corrected using a secondary antibody-only control and staining of PHGDH knockout cell lines.

### Mass spectrometry metabolite measurement

Cells were extracted in ice cold 1:4:5 water:methanol:chloroform with valine-D8 as an internal standard. The aqueous layer was dried under N_2_ and analyzed by LC/MS using a Q Exactive benchtop orbitrap mass spectrometer (Thermo Fisher Scientific, San Jose, CA, USA) equipped with a heated electrospray ionization (HESI) probe, coupled to a Dionex UltiMate 3000 UPLC system (Thermo Fisher Scientific, San Jose, CA, USA). For analysis, dried metabolites were resuspended in 100 μl water, and 1 μl of each sample was separated on a ZIC-pHILIC 2.1 × 150 mm (5-μm particle size) column (EMD) at a flow rate of 0.150 ml/min as follows: 0 to 20 min: linear gradient from 80% to 20% B; 20 to 20.5 min: linear gradient from 20% to 80% B; and 20.5 to 28 min: hold at 80% B. Buffer A was 20 mM ammonium carbonate, 0.1% ammonium hydroxide; buffer B was acetonitrile. Metabolites were detected by mass spectrometry operating in full-scan, polarity switching mode with the spray voltage set to 3.0 kV, the heated capillary held at 275°C, and the HESI probe held at 350°C. The sheath gas flow was set to 40 units, the auxiliary gas flow was set to 15 units, and the sweep gas flow was set to 1 unit. The MS data acquisition was performed in a range of 70 to 1,000 *m*/*z*, with the resolution set at 70,000, the AGC target at 10^6^, and the maximum injection time at 80 ms. Relative quantitation of polar metabolites was performed with XCalibur QuanBrowser 2.2 (Thermo Fisher Scientific, San Jose, CA, USA) using a 5 ppm mass tolerance and referencing an in-house library of chemical standards.

### Size exclusion chromatography

Either recombinant His-PHGDH or recombinant untagged PHGDH was analyzed using a GE HiPrep Sephacryl S-200 HR column. The column was run at 0.5 ml/min for one-column volume in storage buffer. Fractions were collected from just before the void volume to the elution volume of a 17-kDa standard. Fractions were then subjected to SDS-PAGE, and the gels were stained with colloidal Coomassie. After destaining, the gel was imaged and quantitated as described above.

### Electron microscopy and image processing

Specimens were prepared using 12 ng/μl His-PHGDH and 15 ng/μl untagged PHGDH with 0.5 mM NADH by adsorbing 5 μl for approximately 1 min to carbon-coated EM grids that had been glow discharged immediately before use, negative staining with 2% uranyl acetate, sandwiching under a second layer of carbon film, blotting, and drying [24]. On an FEI Tecnai Spirit BioTwin microscope (FEI Tecnai, Hillsboro, OR, USA) with a tungsten filament and an AMT XR16 CCD detector, 103 images of the untagged PHGDH preparation and 107 images of the His-PHGDH preparation were acquired at 0.5- to 1.0-μm defocus (assessed using CTFFIND3 [25] implemented in SPIDER [26]). Identical microscope and camera settings were used for imaging both specimens: ×60,000 magnification (1.84 Å/pixel at the specimen level), 80-kV acceleration voltage, and 1.6-s exposure.

Particles were selected with e2boxer from eightfold binned, band-pass-filtered images. The 23,568 PHGDH particles and 30,894 His-PHGDH particles were cut from the original full-sized images into windows of 160 × 160 pixels using EMAN2 [27]. In preparation for processing, particle images were high-pass filtered, outlier densities median filtered, downsampled fivefold to 32-pixel squares of 9.20 Å/pixel, and normalized such that background densities were scaled to 0 ± 1. Particles from each dataset were aligned, clustered, and realigned within clusters with SPARX using identical reference-free alignment (sxali2d) and k-means clustering (sxkmeans) parameters [28]. To sort averages according to particle size, averages were binarized at a threshold (0.25) that distinguishes the background gray values from the foreground particle densities. Distributions of particle sizes were plotted with Gnuplot. Kolmogorov-Smirnov statistical tests for size distributions of the two samples were calculated using the stats module of SciPy and corroborated with Prism (GraphPad Software).

## Results

### Untagged PHGDH, but not N-terminally tagged PHGDH, can rescue cell proliferation following PHGDH knockdown

Knockdown of PHGDH expression using any of several different small hairpin RNAs impairs proliferation of *PHGDH*-amplified cell lines [3,4]. However, even when multiple hairpins are used, off-target effects can complicate interpretation of results from RNA interference experiments [29]. To confirm that loss of PHGDH expression decreases proliferation of PHGDH-amplified cells, we introduced silent mutations into the PHGDH cDNA to render it resistant to shRNA-mediated knockdown. Initially, an N-terminal FLAG-His epitope tag was included for ease of protein detection and purification. To determine whether FLAG-His-PHGDH expression can rescue shRNA-mediated knockdown of endogenous PHGDH expression, T.T. cells were engineered to express either shRNA-resistant PHGDH cDNA or empty vector as a control. Proliferation of T.T. cells, an esophageal cancer cell line with *PHGDH* copy number gain, has been shown previously to be sensitive to stable knockdown of PHGDH expression [3]. Consistent with previous studies, stable knockdown of PHGDH in control T.T. cells impaired proliferation (Figure 1B). However, exogenous expression of tagged PHGDH failed to rescue this decrease in cell proliferation despite the presence of tagged PHGDH protein (Figure 1B,C). To determine whether exogenously expressed PHGDH failed to rescue proliferation because the epitope tag interfered with protein function, T.T. cells were engineered to express shRNA-resistant PHGDH cDNA lacking an epitope tag (Figure 1D). Expression of PHGDH without an epitope tag rescued proliferation following knockdown of PHGDH (Figure 1E), in agreement with findings in other cells [9]. These findings suggest that introduction of an N-terminal epitope tag impairs the ability of PHGDH to support T.T. cell proliferation.

### PHGDH enzymatic activity is required to rescue cell proliferation following PHGDH knockdown

**Figure 2:**
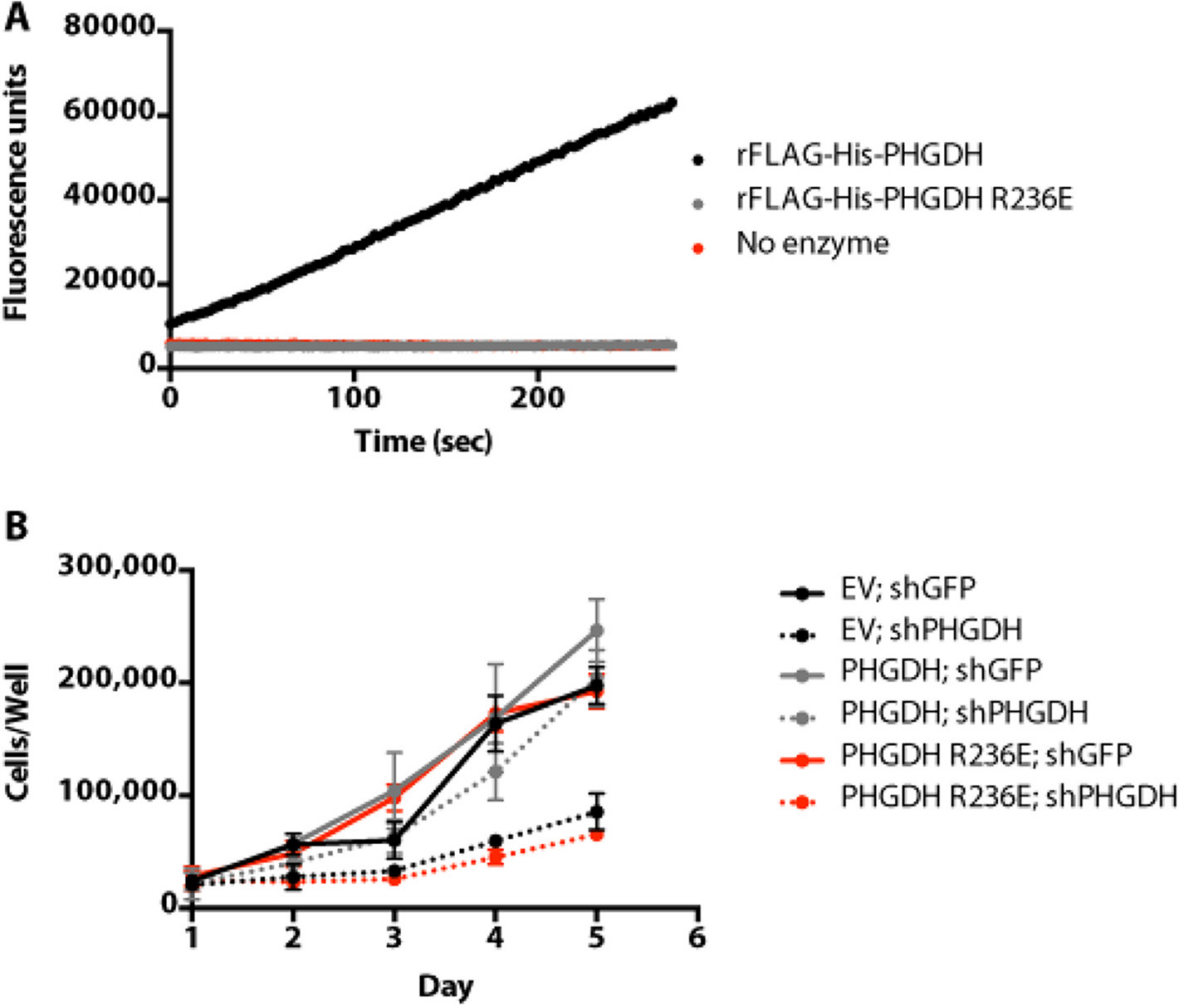
**PHGDH enzymatic activity is required for cell proliferation following PHGDH knockdown. (A)** *In vitro* enzyme activity assessed by tracking NADH production by fluorescence. The assay was performed at saturating substrate concentrations. **(B)** Cell number over time for *PHGDH*-amplified T.T. cells stably expressing shRNA-resistant PHGDH wild type or R236E (enzymatically dead) cDNAs or empty vector (EV) control when infected with virus expressing GFP or PHGDH shRNA. Error bars show standard deviation from the mean.

Despite an inability to rescue proliferation, recombinant FLAG-His-PHGDH exhibits enzyme activity *in vitro* (Figure 2A). PHGDH enzyme activity was measured by tracking NADH production when NAD^+^ and 3-PG were provided at saturating concentrations, with the product PHP removed by coupling the assay to PSAT and providing glutamate (Figure 1A). To confirm that the ability of exogenous PHGDH to rescue endogenous PHGDH knockdown is dependent on enzymatic activity, we generated a mutant that was predicted to be enzymatically inactive. The positively charged arginine residue at position 236 of PHGDH is thought to anchor the carboxyl group of 3-PG. R236 in human PHGDH is analogous to R240 in *E. coli* PHGDH, which when replaced with an alanine was effective at disrupting bacterial enzyme function [16]. Thus, we determined whether mutation of R236 in human PHGDH to a negatively charged glutamate would disrupt enzyme function. FLAG-His-PHGDH R236E was purified from *E. coli* and had no enzyme activity *in vitro* (Figure 2A). We stably overexpressed an shRNA-resistant, untagged version of the R236E mutant in T.T. cells and found that it was incapable of rescuing cell proliferation following knockdown of endogenous PHGDH (Figure 2B and Additional file 1: Figure S1). These data support PHGDH enzymatic function being required for the proliferation of T.T. cells.

### Purification of untagged PHGDH

**Figure 3:**
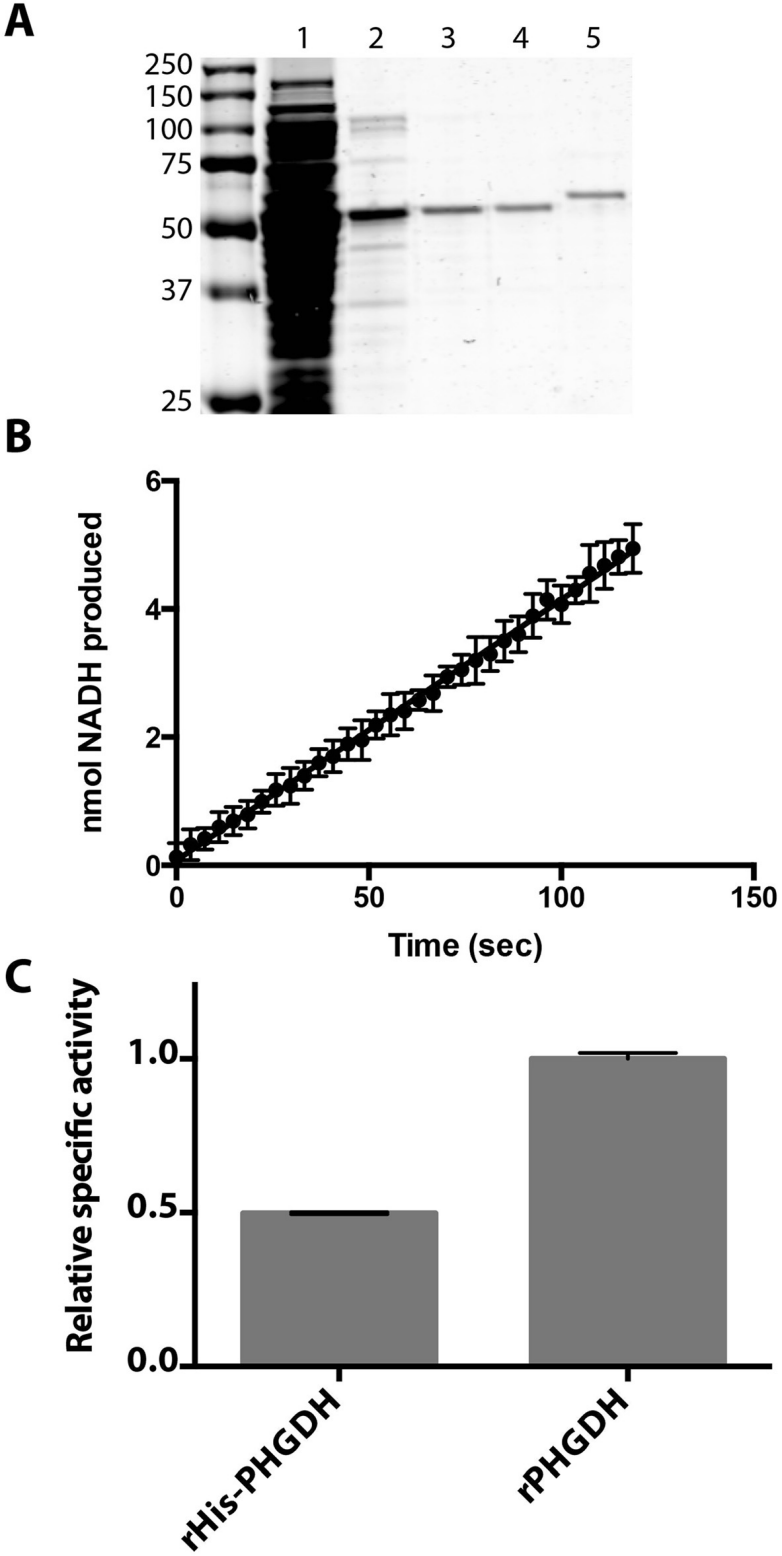
**Purified untagged recombinant PHGDH has higher specific activity than N-terminally tagged PHGDH. (A)** Coomassie gel of rPHGDH (untagged) after each step of the purification: 1. cleared lysate from *E. coli* expressing rPHGDH protein, 2. eluate from Cibacron Blue F3GA column, 3. eluate from anion exchange column, 4. eluate from size exclusion column, and 5. rFLAG-His-PHGDH purified by sequential nickel and size exclusion columns, for comparison. Molecular weight standards in kilodaltons are present to the left of lane 1. **(B)** *In vitro* enzyme activity of untagged rPHGDH. Assay was performed at saturating substrate concentrations. **(C)** Comparison of relative specific activity of rPHGDH and rHis-PHGDH. Error bars show standard deviation from the mean.

To study the differences between tagged and untagged PHGDH *in vitro*, we developed a method to purify the untagged protein. Dye-based pseudoaffinity ligands have been useful for purification of many other enzymes [30,31]. An evaluation of dye-based ligands that bound other enzymes that use NAD(H) as a co-substrate identified Cibacron Blue F3GA as a pseudoaffinity ligand capable of enriching PHGDH protein expressed in *E. coli* from other proteins by severalfold (Figure 3A). We then designed a purification scheme involving three sequential chromatography steps: affinity chromatography using F3GA binding, ion exchange chromatography, and size exclusion chromatography. Using this approach, we recover *E. coli*-expressed PHGDH protein to the same approximate level of purity as His-FLAG-tagged PHGDH prepared using Ni-based affinity chromatography and size exclusion chromatography (Figure 3A).

### Untagged recombinant PHGDH has higher specific activity than N-terminally tagged PHGDH

Untagged recombinant protein is active and has linear kinetics in an *in vitro* assay. The absolute specific activity of untagged PHGDH under these conditions is 0.071 ± 0.001 nmol NADH produced/s/μg protein (Figure 3B). This specific activity is twice the specific activity of recombinant His-PHGDH (Figure 3C), which again exhibits about twice the relative specific activity of recombinant FLAG-His-PHGDH (Additional file 2: Figures S2A, B).

### N-terminally tagged and untagged PHGDH display the same intracellular localization

**Figure 4:**
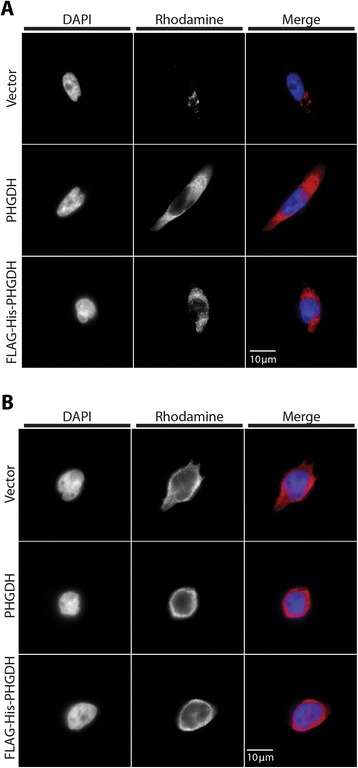
**N-terminal epitope-tagged and untagged PHGDH display the same intracellular localization. (A)** MDA-MB-231 cells, which are PHGDH low, and **(B)** *PHGDH*-amplified T.T. cells expressing either vector control, untagged, or N-terminally tagged PHGDH were stained for PHGDH immunofluorescence.

To determine if the epitope tag disrupts PHGDH localization, MDA-MB-231 cells, which express very little PHGDH [4], and *PHGDH-*amplified T.T. cells were engineered to express vector alone, untagged PHGDH, or FLAG-His-PHGDH and the spatial distribution of PHGDH determined by immunofluorescence. As expected, MDA-MB-231 cells expressing empty vector control showed very little PHGDH staining. In all other samples, PHGDH showed widespread cytoplasmic staining with no difference observed when epitope-tagged PHGDH was introduced (Figure 4A,B). These data are consistent with N-terminally tagging PHGDH having no effect on enzyme localization and argue that an effect on localization does not explain the inability of tagged PHGDH to support cell proliferation.

### Cells expressing N-terminally tagged and untagged PHGDH have an equal 2HG:αKG ratio

In addition to catalyzing 3-PG conversion to PHP, PHGDH from both *E. coli* and humans can also produce D-2-hydroxyglutarate (D-2HG) as a minor product [13,32]. Because D-2HG has been implicated in the pathogenesis of some human cancers we examined whether N-terminally tagged PHGDH results in lower 2HG production than wild-type PHGDH in cells. We measured the 2HG:αKG ratio of T.T. and MDA-MB-231 cells expressing either N-terminally tagged or untagged PHGDH by LC/MS. In both cell lines, 2HG:αKG ratios were not significantly different regardless of which enzyme was expressed (Additional file 3: Figure S3), suggesting that N-terminally tagged PHGDH produces the same amount of 2HG as untagged PHGDH in cells.

### N-terminally tagged and untagged PHGDH exhibit different structural properties by size exclusion analysis

**Figure 5:**
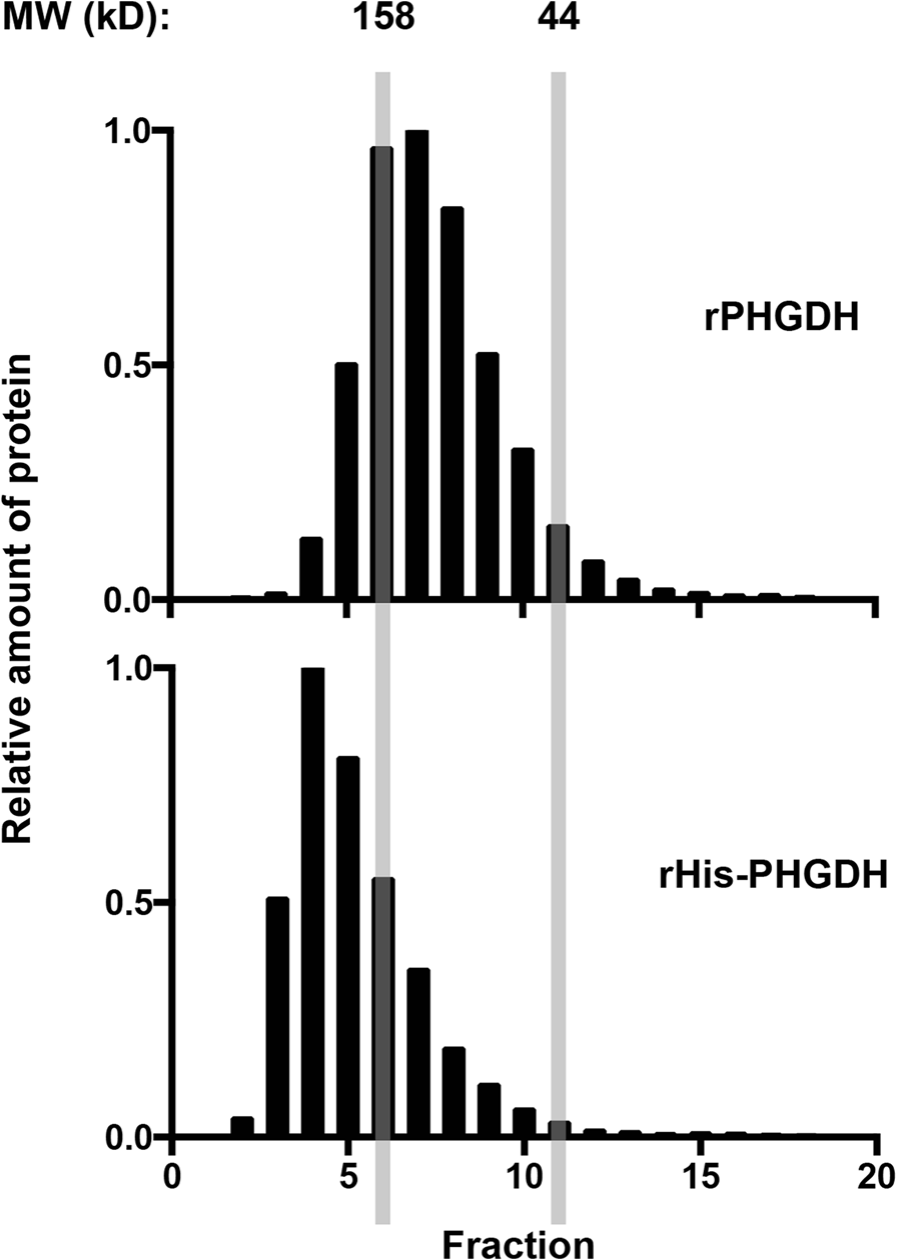
**N-terminal epitope-tagged and untagged PHGDH exhibit different mobility on size exclusion chromatography.** Relative amount of rPHGDH and rHis-PHGDH protein present in fractions eluted from a Sephacryl S-200 size exclusion column. The 158- and 44-kDa standards are shown for comparison, with the column void volume represented in fraction 2.

To interrogate structural differences that might exist between the tagged and untagged recombinant protein, we assessed the mobility of each by size exclusion chromatography. We found that the elution volume for tagged versus untagged PHGDH differed appreciably, with rHis-PHGDH eluting much closer to the void volume of a Sephacryl S-200 HR column (GE Healthcare Bio-Sciences, Pittsburgh, PA, USA), which can resolve globular proteins from 5 to 250 kDa (Figure 5). Comparison with standards of known molecular mass indicates that the peak of untagged PHGDH is approximately 155 kDa, whereas the peak of His-PHGDH is approximately 240 kDa. These data argue that an N-terminal tag affects the mobility of PHGDH on a size exclusion column, and this change in structure may affect the ability of the enzyme to support cell proliferation in some cancer cells.

### N-terminally tagged PHGDH forms larger macromolecular complexes than untagged PHGDH

**Figure 6:**
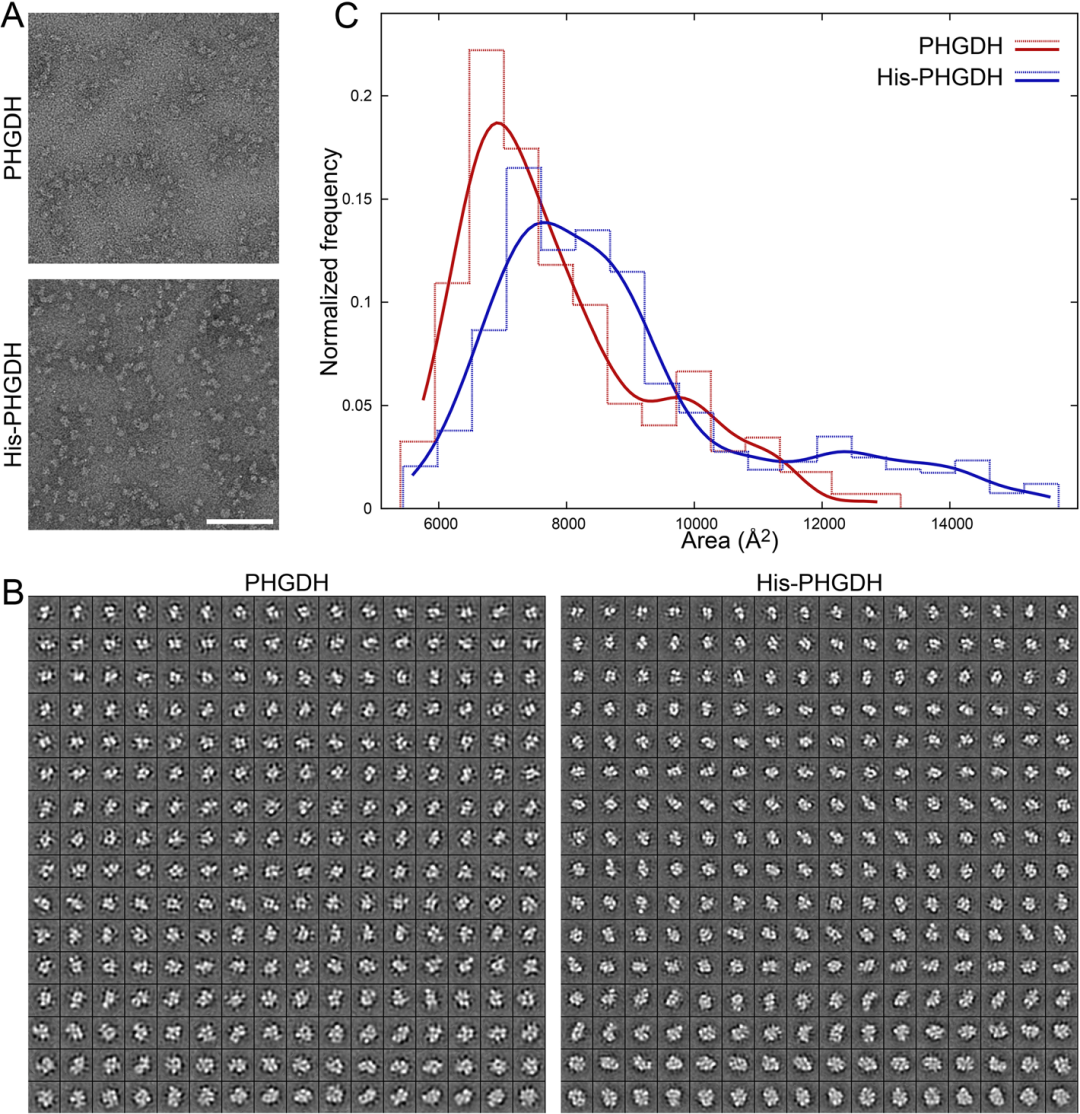
**N-terminally tagged PHGDH forms larger macromolecular complexes than untagged PHGDH. (A)** Representative images obtained from PHGDH and His-PHGDH specimens. Scale bar = 100 nm. **(B)** Montage of class averages of PHGDH and His-PHGDH sorted from smallest (top row) to largest (bottom row). Each average is 294 Å wide at the specimen level. **(C)** Normalized particle-size distributions (particles per bin/total particles) are displayed as histograms (dashed lines) and as smoothed curves (solid lines) for PHGDH (blue) and His-PHGDH (red). The His-PHGDH and PHGDH histograms are offset by 42 Å^2^ to avoid overlapping lines.

To further evaluate potential differences in the structure of the tagged and untagged recombinant protein, negatively stained specimens of each purified protein were prepared and imaged by transmission electron microscopy and protein particles were analyzed by standard methods for alignment and clustering. Comparing the class averages from the PHGDH and His-PHGDH specimens suggests that both preparations have overlapping macromolecular sizes and shapes (Figure 6A,B). To quantitatively assess whether the distribution of particle sizes are different, the area of each average was calculated and compared between PHGDH and His-PHGDH (Figure 6C). The His-PHGDH particle size distribution is significantly shifted toward more massive species (*P* value <0.0001) with a substantial population of particles of size greater than 11,000 Å^2^. The altered distribution is reflected in increased mean particle size of His-PHGDH (8,922 Å^2^) relative to PHGDH (7,855 Å^2^). Given the estimated masses of the complex from size exclusion chromatography, these areas are about twice what would be expected for a dimer, trimer, or tetramer, suggesting that the oligomeric structures of human PHGDH are not approximated well by a sphere. This data demonstrates directly that an N-terminal epitope tag affects the PHGDH structure, which may in turn influence the activity of the protein *in vitro* and the ability to support proliferation after knockdown of the endogenous enzyme.

## Discussion

We demonstrate that N-terminally tagged PHGDH is not functionally equivalent to endogenous PHGDH. Epitope tags have been used for more than 25 years [33-36] and are useful for both purification of recombinant proteins and protein detection when antibodies to an endogenous protein are not available. Although many tags are well tolerated [21], introducing an exogenous peptide sequence onto the N-terminus of PHGDH disrupts protein structure and function. High-quality antibodies for PHGDH are available, and a scheme for PHGDH purification using commercially available materials is described here that renders the use of epitope tags on PHGDH unnecessary.

N-terminally tagged PHGDH not only retains some enzymatic activity *in vitro* but also exhibits structural differences from the endogenous protein; understanding how the tag disrupts PHGDH structure and function could provide new insight into how this protein supports cell proliferation. There is no atomic-resolution structure of the full-length human PHGDH protein, and more detailed structural information would aid in understanding why an N-terminal tag perturbs PHGDH function. PHGDH enzymes from various organisms are grouped into three types (types I to III) based on differences in C-terminal domains thought to be involved in enzyme regulation [37]. All type I and II PHGDH enzymes examined, including *E. coli* [17], *M. tuberculosis* [38], chicken [15], rat [39], and rabbit [40] form tetramers. The inter-subunit interfaces in both type I and II enzymes contain contacts involving the C-terminal regulatory domains. As a result, type III enzymes that lack a C-terminal domain form dimers [37,38]. Human PHGDH is a type I enzyme and is therefore predicted to function as a tetramer; however, the only human PHGDH structure is of a truncated protein lacking the C-terminal domains and thus crystallized as a dimer (PDB 2G76, unpublished). Despite dimeric type III enzymes retaining catalytic function, all plant and animal PHGDH enzymes characterized so far are type I [18,41], suggesting that the C-terminal regulatory domains are important for PHGDH function in these cells.

Few full-length type I and II PHGDH protein structures have been solved; however, examples of both are available. The *E. coli* protein is an example of a type II PHGDH with a known structure and is a symmetrical dimer of dimers in which all monomers assume an identical configuration. In *E. coli* PHGDH, one dimer interface is formed by two adjacent nucleotide binding domains and the other is formed by two C-terminal regulatory domains [17] (PDB 1YBA). The N-terminus of this PHGDH protein appears to be in a position that would accommodate an epitope tag without disrupting the core protein structure. *Mycobacterium tuberculosis* PHGDH is an example of a type I protein [38] (PDB 1YGY). In contrast to the *E. coli* protein, *M. tuberculosis* PHGDH is a tetramer formed by PHGDH monomers with identical amino acid sequence but two different tertiary structures. In the two monomer conformations, the regulatory domains are rotated by 180°. This results in inter-subunit interfaces that involve more complex inter-subunit contacts than the *E. coli* structure [38]. Human PHGDH is also a type I protein, suggesting that its structure may be more similar to *M. tuberculosis* than *E. coli*. In the *M. tuberculosis* structure, the N-terminus is not directly involved in any of the subunit-subunit interfaces, but the increased complexity of the interfaces may account for why placing a tag in this position on the human protein affects structure and function.

There remain a few possibilities for what PHGDH function is disrupted by the N-terminal tag. A threshold of PHGDH activity may be required to support proliferation, and despite retaining canonical enzyme activity, the untagged version is less active and may not be able to attain a level of activity required by proliferating cells. Alternatively, PHGDH could have a non-canonical activity that has not been fully appreciated. The only known PHGDH enzyme activity outside of serine synthesis is an ability to generate small amounts of D-2HG. Because N-terminally tagged PHGDH generates the same 2HG:αKG ratio as untagged PHGDH, it is unlikely that a difference in 2HG production is responsible for the inability of tagged enzyme to support proliferation. Furthermore, the inability of tagged PHGDH to support cell proliferation despite production of similar 2HG levels as the wild-type enzyme, even in MDA-MB-231 cells, which express little to no endogenous PHGDH, also argues against 2HG production as the sole explanation for how increased PHGDH contributes to cancer. If another unknown activity of PHGDH is necessary to support proliferation, our results imply that it must also be lost in the R236E mutant.

PHGDH has been proposed as a potential drug target in cancer [42], and the impact of an epitope tag has practical implications for designing screens for small-molecule PHGDH inhibitors. Because tagged PHGDH retains some enzyme activity, the convenience of generating recombinant tagged protein might result in the use of this protein for drug screening. Whether this will identify molecules that impair proliferation of PHGDH-dependent cells remains to be determined, but the use of recombinant untagged protein is preferred based on our findings. If small molecules are identified using either approach, they could provide insight into whether the ability to inhibit 3-PG to PHP conversion alone is the relevant PHGDH function in cells and help understand the role of this enzyme in cell proliferation and human cancer.

## Conclusions

Addition of an N-terminal epitope tag to PHGDH alters enzyme structure, reduces catalytic activity, and abrogates its ability to support proliferation of PHGDH-dependent cancer cells. A simple and effective scheme for purification of recombinant untagged PHGDH is presented, making use of epitope tags to generate purified enzyme unnecessary.

## Additional files

Additional file 1: Figure S1. Western blot analysis of PHGDH expression in the T.T. cells shown in Figure 2B. Additional file 2: Figure S2.
*In vitro* enzyme activity and relative specific activity of rHis-PHGDH and rFLAG-His-PHGDH. (A) *In vitro* enzyme activity of rHis-PHGDH and rFLAG-His-PHGDH was assessed as in Figure 2A. (B) Comparison of relative specific activity of rHis-PHGDH and rFLAG-His-PHGDH. Error bars show standard deviation from the mean. Additional file 3: Figure S3. Relative intracellular 2HG:αKG ratio as measured by LC/MS. Error bars show standard error of the mean. *P* values were calculated by a two-tailed Student’s *t*-test.

## Abbreviations

3-PG: 3-phosphoglycerate
D-2HG: D-2-hydroxyglutarate
IMAC: immobilized metal ion affinity chromatography
PHGDH: phosphoglycerate dehydrogenase
PHP: phosphohydroxypyruvate
PSAT: phosphoserine aminotransferase
P-Ser: phosphoserine
PSPH: phosphoserine phosphatase
SHMT: serine hydroxymethyltransferase
TCA cycle: tricarboxylic acid cycle
αKG: alpha-ketoglutarate
