# Supplementary figures and images for: An epitope tag alters phosphoglycerate dehydrogenase structure and impairs ability to support cell proliferation
Source: Cancer Metab. 2015 Apr 29;3:5. doi: 10.1186/s40170-015-0131-7 (PMC4414297; doi:10.1186/s40170-015-0131-7)

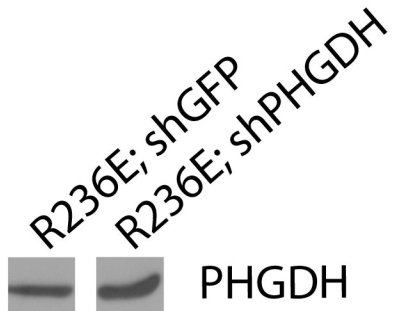

**Mattaini et al., Suppl. Figure 1**

Supplement: Additional file 1: Figure S1. — Western blot analysis of PHGDH expression in the T.T. cells shown in Figure 2B. [file 40170_2015_131_MOESM1_ESM.pdf]

A

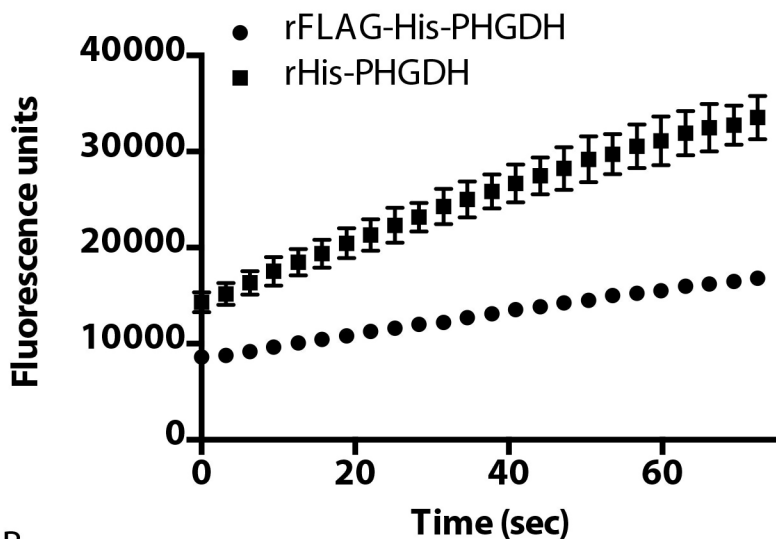

B

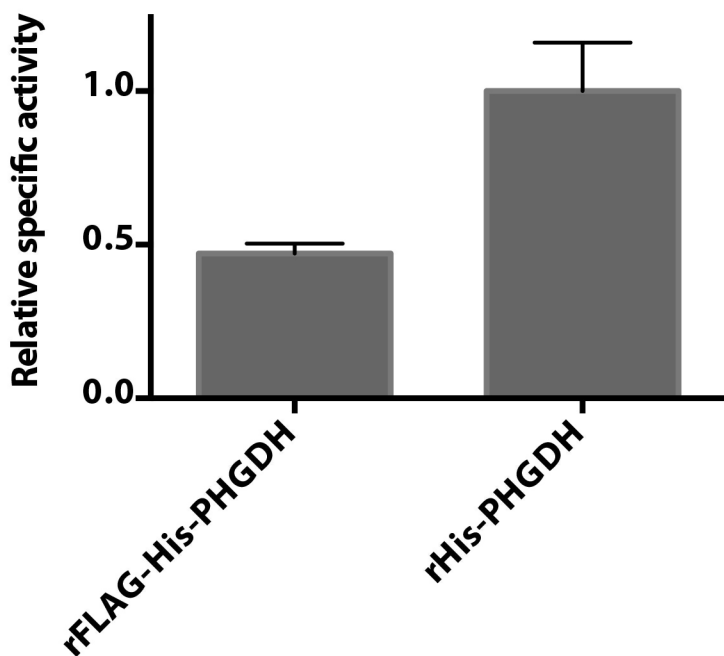

Supplement: Additional file 2: Figure S2. — In vitro enzyme activity and relative specific activity of rHis-PHGDH and rFLAG-His-PHGDH. (A) In vitro enzyme activity of rHis-PHGDH and rFLAG-His-PHGDH was assessed as in Figure 2A. (B) Comparison of relative specific activity of rHis-PHGDH and rFLAG-His-PHGDH. Error bars show standard deviation from the mean. [file 40170_2015_131_MOESM2_ESM.pdf]

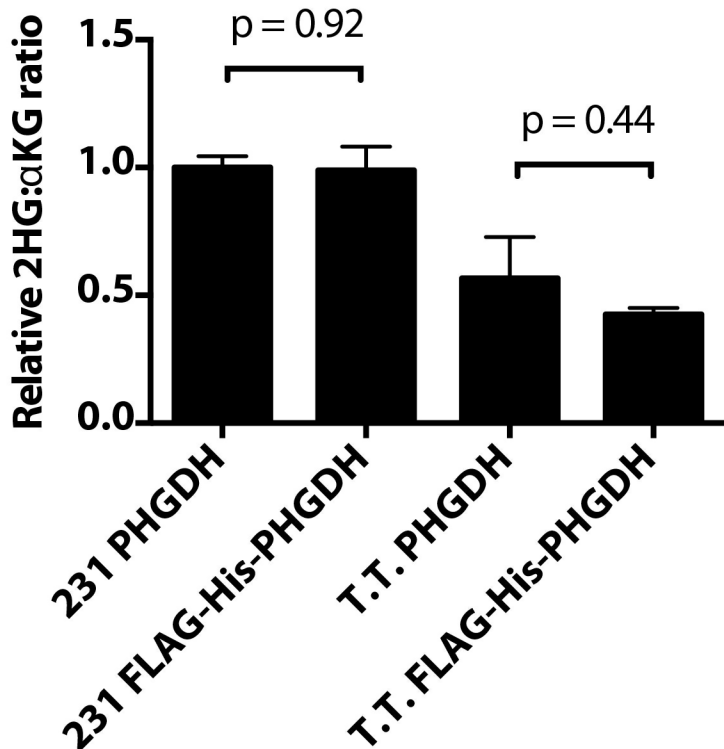

**Mattaini et al., Supplemental Figure 3**

Supplement: Additional file 3: Figure S3. — Relative intracellular 2HG:αKG ratio as measured by LC/MS. Error bars show standard error of the mean. P values were calculated by a two-tailed Student’s t-test. [file 40170_2015_131_MOESM3_ESM.pdf]
